# How Well Do Randomized Controlled Trials Reflect Standard Care: A Comparison between Scientific Research Data and Standard Care Data in Patients with Intermittent Claudication undergoing Supervised Exercise Therapy

**DOI:** 10.1371/journal.pone.0157921

**Published:** 2016-06-23

**Authors:** S. Dörenkamp, E. P. E. Mesters, M. W. G. Nijhuis-van der Sanden, J. A. W. Teijink, R. A. de Bie, T. J. Hoogeboom

**Affiliations:** 1 Department of Epidemiology, Functioning and Rehabilitation Programme, CAPHRI School for Public Health and Primary Care, Maastricht University, Maastricht, The Netherlands; 2 Radboud University Medical Center, Institute for Health Sciences, IQ healthcare and Department of Rehabilitation, Nijmegen, The Netherlands; 3 Department of Vascular Surgery, Catharina Hospital, Eindhoven, The Netherlands; Maastricht University Medical Center, NETHERLANDS

## Abstract

**Objective:**

The aim of the present study was to assess the degree and impact of patient selection of patients with intermittent claudication undergoing supervised exercise therapy in Randomized Controlled Trials (RCTs) by describing commonly used exclusion criteria, and by comparing baseline characteristics and treatment response measured as improvement in maximum walking distance of patients included in RCTs and patients treated in standard care.

**Methods:**

We compared data from RCTs with unselected standard care data. First, we systematically reviewed RCTs that investigated the effect of supervised exercise therapy in patients with intermittent claudication. For each of the RCTs, we extracted and categorized the eligibility criteria and their justifications. To assess whether people in RCTs (n = 1,440) differed from patients treated in daily practice (n = 3,513), in terms of demographics, comorbidity and walking capacity, we assessed between group-differences using t-tests. To assess differences in treatment response, we compared walking distances at three and six months between groups using t-tests. Differences of ≥15% were set as a marker for a clinically relevant difference.

**Results:**

All 20 included RCTs excluded large segments of patients with intermittent claudication. One-third of the RCTs eligibility criteria were justified. Despite, the numerous eligibility criteria, we found that baseline characteristics were largely comparable. A statistically significant and (borderline) clinically relevant difference in treatment response after three and six months between trial participants and standard care patients was found. Improvements in maximum walking distance after three and six months were significantly and clinically less in trial participants.

**Conclusions:**

The finding that baseline characteristics of patients included in RCTs and patients treated in standard care were comparable, may indicate that RCT eligibility criteria are used implicitly by professionals when referring patients to standard physiotherapy care. The larger treatment response reported in standard physiotherapy care compared to clinical trials, might suggest that scientific studies underestimate the benefits of supervised exercise therapy in patients with intermittent claudication.

## Introduction

*“Can I translate results found in patients enrolled in a randomized controlled trial (RCT) to the patient I am treating in my daily clinical practice*?*”* This is one of the fundamental questions healthcare professionals have to ask themselves when interpreting scientific evidence. Concerns frequently arise as to how well a therapy may perform beyond highly selected patient populations and in different clinical settings than those studied in RCTs [1–5].

Differences between patients participating in RCTs and patients treated in standard care, as well as differences in clinical interventions between RCTs and standard care might affect the effectiveness of a therapy. Randomized controlled trials are only able to guide standard care and clinical decision-making when the research findings can be expected to be similar in other groups of patients not included [6, 7]. This requirement is not always met, which makes it difficult for clinicians to know to what extent the evidence for the effectiveness of an intervention applies to a given patient [8]. In this study, we aim to illustrate the degree of patient selection in RCTs by comparing baseline characteristics of patients included in clinical trials and patients treated in standard physiotherapy care. Furthermore, we aim to describe the differences in treatment response between patients enrolled in RCTs and those treated in standard care. Patients with intermittent claudication undergoing supervised exercise therapy are used as a case example, as our group has ample experience with both intermittent claudication and supervised exercise therapy (helping us to correctly interpret the utilized eligibility criteria) and access to a large, well-documented, unselected database of patients with intermittent claudication undergoing supervised exercise therapy (allowing a direct comparison between scientific research and real-life practice).

We aim to address the following three research questions:

1. Which patients with intermittent claudication are excluded in RCTs studying the effect of supervised exercise therapy and what are the reasons for excluding these patients?
2. To what extent are baseline characteristics of patients with intermittent claudication included in RCTs and patients with intermittent claudication treated with supervised exercise therapy in standard care comparable?
3. To what extent are supervised exercise therapy treatment responses (in terms of maximum walking distance) after three and six months of patients included in RCTs and patients treated in standard physiotherapy care comparable?

We hypothesized that, due to a strict selection of patients with intermittent claudication participating in RCTs (favouring less complex patients), the baseline maximum walking distance and the improvement in maximum walking distance after three and six months of supervised exercise therapy is larger in RCTs as compared to the treatment responses of patients with intermittent claudication treated in standard supervised exercise therapy care.

## Materials and Methods

### Study design

We used the convergent parallel design [9] to compare two separate data sources: 1. data from RCTs investigating the effect of supervised exercise therapy in patients with intermittent claudication and 2. data from patients with intermittent claudication treated with supervised exercise therapy in the context of standard care physiotherapy. Both data sources were extracted and analyzed during the same phase of this study. Using qualitative data on the applied eligibility criteria and their justifications, we studied the extent of patient selection in RCTs. We extracted quantitative data of baseline characteristics and maximum walking distance from RCTs, and compared it with data from standard care physiotherapy to study differences at baseline and in treatment response after three and six months between patients included in RCTs and patients treated in standard care. Both qualitative and quantitative data were combined to formulate an overall interpretation of the results. To enhance transparency and reproducibility this article is written according to the Preferred Reporting Items for Systematic reviews and Meta-Analysis (PRISMA) presented in S1 PRISMA Checklist [10].

### Systematic review

#### Information sources and search

We performed a systematic search of articles from scientific medical journals published in the electronic online databases MEDLINE (PubMed), Embase (Ovid) and Cochrane Library up to mid-August 2015. The search strategy was developed by SD and TH and included MeSH terms and keywords and is presented in S1 Table. To identify additional articles, all reference lists were checked for further relevant citations. We compared trials reporting information on the same set of patients and only included those with the most complete information on the patients’ baseline characteristics and treatment response.

#### Study selection

Studies were eligible for inclusion if: 1. the design was an RCT comparing supervised exercise therapy in patients with symptomatic peripheral arterial disease with standard care and/or other exercise recommendations. We focus on RCTs only, because they are accepted as the most unbiased measure of the efficacy of an intervention. Only RCTs comparing supervised exercise therapy with standard care and/or any other exercise recommendation and not RCTs comparing supervised exercise therapy with supervised exercise therapy with some kind of augmentation to it are included, because these effectiveness trials (using two treatments that lie within the range of usual-care practices) are the RCTs that are typically used to communicate the effectiveness of supervised exercise therapy to stakeholders. Due to the fact that we inquire to evaluate the effectiveness of supervised exercise therapy only effectiveness trials are included; 2. Peripheral arterial disease was diagnosed by the Ankle-Brachial Index (ABI) <0.9 in rest and/or <0.73 after exercise, because these are the criteria for patients to enter physiotherapy care; 3. the duration of supervised exercise therapy was at least three months, so as to be able to compare supervised exercise therapy treatment response between RCTs and standard care [11]; 4. the authors reported maximum walking distance or time measured by a graded treadmill test so that the outcome measure between RCT and standard care is equivalent or can be recalculated to become equivalent. All citations were independently screened and reviewed by SD and TH using the online software for intervention reviews Covidence supported by Cochrane [12]. We identified RCTs investigating the effect of supervised exercise therapy in patients with intermittent claudication on the basis of title and abstract. A more detailed inquiry based on full-text screening ensured that only articles fulfilling the eligibility criteria mentioned above were included in the meta-analysis.

#### Data collection process and data items

To evaluate eligibility criteria used and reasons for excluding specific patients, we listed first author, publication year and eligibility criteria per trial. In a second table, we recorded justifications for eligibility criteria applied per RCT. Information on eligibility criteria and justifications for eligibility criteria used was extracted in duplicate by SD and TH.

To extract relevant data on baseline characteristics we used a self-designed, standardized data extraction form. Data about the author, the publication year, the country in which the RCT was conducted, the mean age (years), the percentage of males, the mean body mass index (kg/m^2^), the percentage of smokers and mean baseline maximum walking distance in meters of the supervised exercise therapy group was listed by TH and checked by SD. Only data from the supervised exercise therapy group of RCTs was used, because the aim of the present study is to compare research data with patients treated with supervised exercise therapy in standard care physiotherapy.

We extracted data on treatment outcome using a second self-designed standardized data sheet. Data extraction was done by SD and controlled by TH. We obtained exercise training and testing parameters (exercise type, exercise length, exercise duration, exercise frequency, walking speed, presence/absence of supervision and treadmill test protocol). In addition, we listed the number of patients that received supervised exercise therapy as well as mean maximum walking distance in meters after three and six months. Maximum walking distance presented in time (minutes or miles per hour) as well as maximum walking distance recorded in feet was converted into meters [13, 14].

#### Risk of bias across studies

We assumed that summary statistics on treatment outcome, defined as maximum walking distance, were adequately reported in all 20 RCTs. A normal distribution of data was assumed to be present in trials reporting mean maximum walking distance and standard deviation (SD) and a skewed data distribution was supposed to be present in trials reporting median and Interquartile range (IQR). Because in a skewed distribution the mean is pulled in the direction of extreme scores (tail), inadequate use of the summary statistic mean in RCTs included may have introduced over-reporting of maximum walking distance in case of right skewed data and under-reporting of maximum walking distance in case of left skewed data.

#### Quality assessment

The Physiotherapy Evidence Database PEDro stores RCTs conducted in the field of physiotherapy research. This database is updated once a month. The methodological quality of all RCTs stored in the database is rated with the PEDro scale by the well-trained staff of the Centre for Evidence-Based Physiotherapy. Results of the assessment of the methodological quality of trials are also published in the PEDro database. To ensure the quality of ratings all raters underwent training; all RCTs are rated twice; a third rater resolved possible disagreements; informal and non-systematic checks of the quality of some ratings were performed; and the opportunity for PEDro users to dispute trial ratings is offered.

The PEDro scale is a checklist of 11 scored questions (yes or no) rating the methodological quality of RCTs. Criteria focus on the external validity (criterion 1), the internal validity (criterion 2–9) and the statistical information provided (criterion 10 and 11). A ‘yes’ is only awarded when a criterion is clearly satisfied. Each satisfied item contributes one point to the total PEDro score (0 to 10 points), except for item 1 which is the only item related to the external validity of the trial. The PEDro scale is based on a Delphi list developed by Verhagen et al. [15] Results of previous research show that the PEDro scale is reliable for use in systematic reviews of physiotherapy RCTs [16]. We found information about the methodological quality of all RCTs included in the present study in PEDro, except for Mays et al. [17], a very recent RCT. The methodological quality of this RCT was rated in duplicate (by SD and TH) using the PEDro scale. No disagreements between both raters occurred.

### Standard physiotherapy care data

We used data from patients with a physician-confirmed symptomatic peripheral arterial disease (ABI <0.9 at rest) who were treated with supervised exercise therapy in standard care physiotherapy between 2006 and 2011. Patient data were prospectively collected by 197 physiotherapists using Electronic Medical Records. We did not include data of patients without a baseline measurement in the present study, because one aim was to compare baseline characteristics of patients enrolled in trials with baseline characteristics of patients treated in standard care physiotherapy.

Data of patient characteristics recorded in the electronic medical record include information on age, gender, BMI, smoking behavior and comorbidity. The BMI of each patient was calculated according to the clinical guideline on the Identification, Evaluation and Treatment of Overweight and Obesity in adults [18]. Smoking behavior was recorded as current smoker or current non-smoker. The presence or absence of cardiovascular comorbidity, pulmonary comorbidity, internal comorbidity, orthopedic comorbidity and neurologic comorbidity was recorded according to the standard hospital registration. Diseases belonging to all categories of comorbidity are listed in S2 Table. Patients were treated with supervised exercise therapy according to the evidence-based guideline intermittent claudication recommendation of the Royal Dutch Society for Physical Therapy [19]. The primary outcome measurement of the therapy was improvement in maximum walking distance. To measure maximum walking distance, physiotherapists performed a standardized graded treadmill test according to the treadmill test protocol described by Gardner et al [20]. All data was gathered in the context of standard care physiotherapy. The ethics committee/institutional review board 'Medisch Ethische Toetsingscommissie van Atrium Medisch Centrum, Orbis Medisch en Zorgconcern en Zuyd Hogeschool' approved at August, 19th 2013 the physiotherapy cohort study. Data was gathered in the context of standard care. All participants signed written informed consent for their clinical records to be used and patient records/information was anonymized and de-identified prior to analysis (METC number: 13-N-85).

### Data analysis

Because inclusion and exclusion criteria are interchangeable by merely adding a ‘not’ to any given criterion, we studied both to receive a complete picture of the eligibility criteria applied. To evaluate which eligibility criteria were applied in the included RCTs, SD and TH first independently listed all eligibility criteria per RCT. Subsequently, both researchers in consultation categorized each of the inclusion and exclusion criteria. The following categories were created: ‘patient characteristics’, ‘unstable medication use’, ‘comorbidity’, ‘walking capacity’ and ‘other criteria’. Both assessors independently counted the number of trials in which the criteria per category occurred; counts and percentages were listed. Finally, both researchers checked whether and how often justifications for applied eligibility criteria were provided. With regard to comorbidity, the percentage of RCT participants with comorbidity was noted per RCT. Comorbidity reported in RCTs was often disease-specific. To be able to compare RCT participants with standard care physiotherapy patients with regard to comorbidity, we categorized disease-specific comorbidities listed in the RCTs into the categories of comorbidity that were used in the standard physiotherapy care EMR data, namely: cardiovascular, pulmonary, internal, orthopedic and neurologic comorbidity. The disease-specific comorbidity reporting in RCTs did not allow us to estimate the percentage of participants with comorbidity per category due to possible double-counts of participants. Therefore, we calculated a worst-case and a best case-scenario. In the worst-case scenario, disease-specific cases of comorbidity were added up per RCT and per category. In the best-case scenario, the highest percentage comorbidity per category and RCT was listed. We compared both scenarios with the percentage of comorbidity in standard care patients. For the sake of clear presentation in the text, the average percentage between worst-case and best-case scenario will be presented.

To compare baseline characteristics of patients included in RCTs and patients seen in standard physiotherapy care, we first pooled relevant baseline characteristics (age, sex, BMI, smoker (yes/no), and maximum walking distance) of all individual trials according to the methods described in the Cochrane Handbook [21]. Second, we tested whether the pooled estimates from the RCTs were significantly different from the estimates of the same baseline characteristics of the standard care physiotherapy cohort, using t-tests. In addition, we depicted mean maximum walking distance and their respective SDs per study and for the cohort in a forest plot to allow visual comparison. To investigate whether treatment response after supervised exercise therapy differs between treatment in standard care and treatment in RCTs, we described the differences in maximum walking distance in meters after three and six months. Median maximum walking distance after three and six months of standard care was compared with pooled estimates of the treatment response measured in RCTs using a t-test. Furthermore, a difference in maximum walking distance between patients included in RCTs and patients treated in standard care of ≥15% was set as a marker for a clinically relevant difference [22]. We performed sensitivity analysis to take care of different treadmill protocols used to assess maximum walking distance in different RCTs.

## Results

Overall, RCTs provided data about 1,440 patients, and we used data of 3,531 patients from standard care physiotherapy. First, we present the results of our systematic review on RCTs investigating the effect of supervised exercise therapy in patients with intermittent claudication. Then, we show information about the eligibility criteria applied in RCTs and reasons for exclusion, followed by the results of our comparison of RCT data and standard care data at baseline and after three and six months.

### Study selection

**Fig 1:**
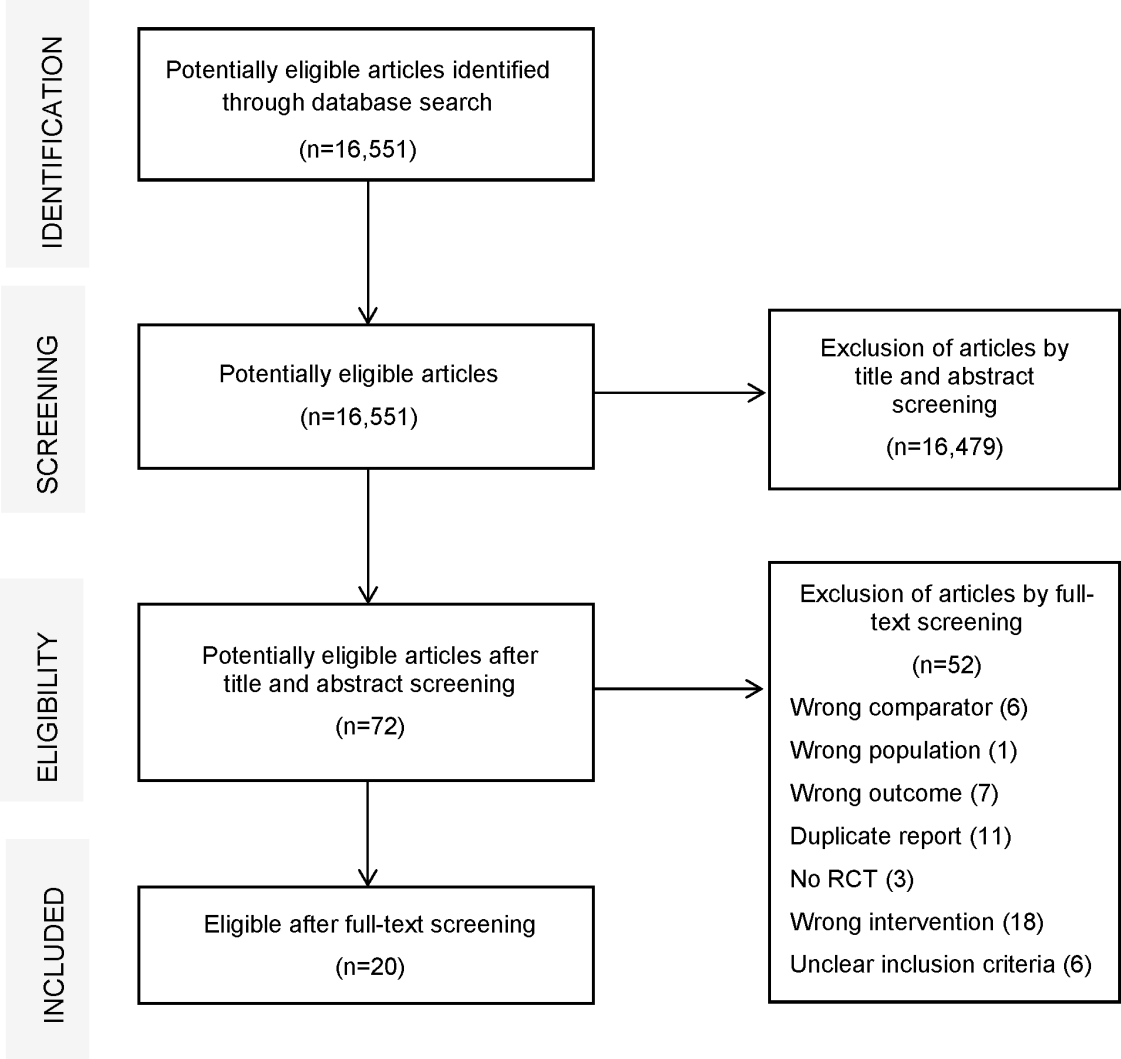
Flow diagram of screened, eligible and included randomized controlled trials.

The systematic search identified 16,551 potentially eligible articles in total, and 72 full-text RCTs were recognized for possible inclusion. During full-text screening, we excluded RCTs for the following reasons: a comparator other than any exercise recommendation or ‘doing nothing’ (12%); inappropriate patient group(s) (14%); maximum walking distance or walking time was not reported (14%); a nonrandomized design (6%); or supervised exercise therapy was not the intervention (35%). After identification of duplicate reports (21%), 20 eligible RCTs remained. A flow diagram of screened, eligible and included randomized controlled trials is presented in Fig 1 [17, 23–41].

### Methodological quality

Total PEDro quality scores varied from 3 to 7 out of a maximum of 10 points. In all RCTs bias occurred due to a lack of blinding of subjects and therapists. In addition, 80% of the trials reported that group allocation was not concealed, 75% had no blinded assessors and 55% of all RCTs did not report information about an intention-to-treat analysis (S3 Table).

### Inventory of eligibility criteria and reasons for exclusion

Patient characteristics were the reason for exclusion in 30% of the RCTs (Table 1). Patients younger than 18 were excluded most often (20% of trials). (Unstable) lipid lowering, anti-platelet and antihypertensive medication use was the reason for exclusion in 65% of the trials. Patients were excluded due to the presence of comorbidity in all trials. Cardiovascular comorbidity (85%) and internal comorbidity (50%) were the main comorbidities for exclusion. Patients that suffer from a pulmonary and/or an orthopedic comorbidity were excluded in 40% of the trials. Impairment in walking capacity was the reason for exclusion in 70% of the RCTs. Being unable to walk on a treadmill with at least a speed of 3.2 km/h (35%) and gait abnormalities that had a different cause than peripheral arterial disease (45%) were most often reported. Other criteria were the reason for exclusion in 55% of the trials. Within this group, vascular surgery, angioplasty, lumbar sympathectomy and lower extremity revascularization in the six months preceding or scheduled at the start of the trial were the main reason for exclusion (70%) (Table 1).

**Table 1:** Summary of eligibility criteria used in all RCTs.

Eligibility criteria	No (%) of RCTs in which each eligibility criterion was applied
**Patient characteristics**	**6 (30)**
Age	
<18 year	4 (20)
>70 year	1 (5)
Sex Female	1 (5)
Women in menopausal status and those taking estrogen	1 (5)
BMI ≥ 30 kg/m^2^	1 (5)
**Unstable medication use**	**13 (65)**
**Comorbidity**	**20 (100)**
Cardiovascular	17 (85)
Pulmonary	8 (40)
Internal	10 (50)
Orthopedic	8 (40)
Neurologic	5 (25)
**Walking capacity**	**14 (70)**
Unable to walk on a treadmill at a speed of at least 3.2 km/h	7 (35)
Gait abnormalities not due to peripheral arterial disease	9 (45)
Maximum walking distance >1600 meter at baseline	1 (5)
Maximum walking distance ≥ 500 meter at baseline	1 (5)
Maximum pain-free walking distance <150 meter	1 (5)
No functional limitation due to IC during treadmill test	3 (15)
**Other criteria**	**11 (55)**
Concurrent supervised exercise therapy	4 (20)
No native speaker	2 (10)
No insurance for supervised exercise therapy	1 (5)
Invasive peripheral arterial disease treatment (preceding 6 months or planned)	14 (70)
Substance abuse	1 (5)
Not living independently	2 (10)
Inability and/or unwillingness to exercise	4 (20)
Inability to obtain ABI	3 (15)
Rest leg pain	4 (20)
Unstable intermittent claudication symptoms (preceding 3 months)	1 (5)

Abbreviations: BMI = body mass index

Regarding the presence of comorbidity, we found that cardiovascular comorbidity (65% vs 43%) and neurologic comorbidity (24% vs 12%) were more prevalent in standard care than in RCTs, respectively. Pulmonary (18% vs 16%), orthopedic (24% vs 20%) and internal comorbidity (54% vs 58%) were comparable between standard care and RCTs, respectively (S4 Table).

Eligibility criteria were justified by authors in 30% of the RCTs [26, 29, 30, 33, 36, 37]. For instance, Gardner et al. [26] referred to the Guidelines of the American college of Sports Medicine [42] and a study conducted by Regensteiner et al. [43] for justification. The other five RCTs [26, 29, 33, 36, 37] reported detailed rationales for the applied eligibility criteria. Most frequent reasons for applied exclusion criteria were: (1) that the response to supervised exercise therapy might be affected by a certain characteristic; (2) that the aim of the study requires exclusion of certain groups of participants; (3) that patients with certain characteristics were not likely to complete supervised exercise therapy; (4) that the characteristic might limit and/or alter walking ability (see S5 Table for details).

### Description and comparison of RCT data and standard care data at baseline

A description of RCT data and standard care data is displayed in Table 2. Supervised treadmill walking, coached by a physiotherapist or another exercise specialist was the type of exercise in nearly all RCTs and in standard physiotherapy care. In trials supervised exercise therapy was performed on average for 22 weeks, three times a week with on average 42 minutes per exercise session. In standard physiotherapy care supervised exercise therapy was performed for on average 26 weeks, two to three times a week for 30 minutes per exercise session.

**Table 2:** Patient characteristics, parameters of exercise training and Maximum Walking Distance of RCTs (individually), RCTs (cumulatively) and standard care at baseline, 3 months and 6 months of Supervised Exercise Therapy.

	Baseline													3 months		6 months		
	Country	N	Age Mean (SD) (year)	Male (%)	BMI Mean (SD)(kg/m^2^)	Smoker (% yes)	MWD BaselineMean (SD)	Exercisetype	ExerciseLength (wk)	ExerciseDuration (min)	ExerciseFrequency (x per wk)	Walking speed (km/h)	Supervision (Yes/No)	N 3 mo	MWD 3 moMean (SD)	N6 mo	MWD6 moMean (SD)	MWDTest**
Individual RCTs																		
Allen, 2010	USA	33	67 (12)	-	27.8 (5.0)	31	506 (71)	SET	14	40	3	3.2	Yes	15	680(72)	-	-	GS
Crowther, 2008	Australia	21	69 (8)	48	28.8 (4.8)	19	274 (146)	SET	52	40	3	3.2	Yes	-	-	-	-	GS
Cucato, 2013	Brazil	25	63 (7)	100	25.6 (3.1)	24	805 (237)	SET	12	30	2	3.2	Yes	13	1100 (236)	-	-	GS
Gardner, 2002	USA	31	72 (4)	92	29.9 (4.3)	0	451 (199)	SET	76	40	3	3.2	Yes	-	-	17	809 (73)	GS
Gardner, 2011	USA	92	65 (11)	48	29.8 (6.0)	10	363 (201)	SET	12	45	3	3.2	Yes	33	481 (250)	-	-	GS
Gardner, 2012	USA	142	68 (8)	85	28.3 (4.7)	60	373 (212)	SET	26	40	3	3.2	Yes	-	-	80	664 (264)	GS
Gardner, 2014	USA	180	66 (10)	53	29.1 (6.2)	38	356 (218)	SET	12	40	3	3.2	Yes	60	487 (266)	-	-	GS
Hiatt,1990	USA	19	60 (12)	100	-	58	331 (99)	SET	12	50	3	3.2	Yes	10	742 (187)	-	-	Hiatt
Hiatt,1994	USA	29	67 (6)	100	-	70	421 (224)	SET	12	50	3	3.2	Yes	10	785 (390)	10	918 (390)	Hiatt
Hodges, 2008	UK	28	68 (8)	-	26.7 (3.4)	-	355 (230)	SET	12	30	2	3.2	Yes	14	623 (311)	-	-	GS
Kruidenier, 2011	NL	61	62 (10)	61	27.1 (4.2)	56	595 (307)	SET	26	30	2–3	3.2	Yes	32	974 (513)	34	956 (490)	GS
Mays, 2015	USA	20	65 (10)	80	28.5 (5.0)	-	478 (292)	SET	15	35	3	NR	Yes	-	-	-	-	GS
McDermott, 2004	USA	25	70 (9)	52	28.7 (5.3)	19	116 (70)	SET	14	50	3	3.2	Yes	17	178 (89)	-	-	GS
McDermott, 2009	USA	104	70 (11)	47	30.2 (6.7)	14	383 (227)	SET	26	40	3	3.2	Yes	-	-	48	619 (297)	GS
McDermott, 2013	USA	194	70 (10)	50	29.1 (6.7)	24	412 (248)	OGW	26	50	5	NR	No	-	-	86	504 (318)	GS
Mika, 2006	Poland	55	59 (8)	87	-	76	386 (59)	SET	14	50	3	3.2	Yes	27	584 (70)	-	-	Hiatt
Mika, 2011	Poland	61	63 (7)	87	27.7 (3.2)	80	543 (65)	SET	12	55	3	3.2	Yes	30	849 (61)	-	-	GS
Nicolaï, 2010	NL	252	66 (9)	63	27.9 (4.6)	42	260 (153)	SET	52	30	3	3.2	Yes	169	530 (573)	169	610 (692)	GS
Treat-Jacobson, 2009	USA	15	67 (10)	74	27.5 (4.3)	-	432 (255)	SET	12	60	3	3.2	Yes	-	-	-	-	Other
Tsai, 2002	China	53	76 (4)	83	23.3 (2.5)	-	389 (191)	SET	12	30	3	3.2	Yes	27	668 (198)	-	-	GS
**Pooled estimates of RCTs**	**World**	**1,440**	**67 (9)**	**73**	**26.0 (5.0)**	**39**	**382 (203)**	**SET**	**22**	**42**	**3**	**3.2**	**Yes**	**457**	**605 (407)**	**444**	**641 (495)**	**-**
**Standard care**	**NL**	**3,531**	**67 (10)**	62*	26.7* **(4.3)**	**41**	**370 (287)**	**SET**	**26**	**30**	**2–3**	**3.2**	**Yes**	**1,738**	690* **(448)**	**1,252**	800* **(457)**	**GS**

* p< 0.05

Abbreviations: BMI = Body-Mass Index; MWD = Maximum Walking Distance; SD = Standard Deviation; OWG = Over Ground Walking; GS = Gardner-Skinner Protocol; NR = Not Reported

** detailed information about treadmill testing assessment can be found in S6 Table.

The mean (SD) age of patients with IC included in the RCTs and patients with IC that were referred to the physiotherapist for the treatment of supervised exercise therapy was 67 years in both groups. The percentage of males was significantly higher in the trial patients (73%) compared to the standard physiotherapy care patients (62%). The body mass index of patients treated in standard care was significantly higher in patients included in RCTs (26.7 kg/m^2^ vs 26.0 kg/m^2^, *p* < 0.05).

**Fig 2:**
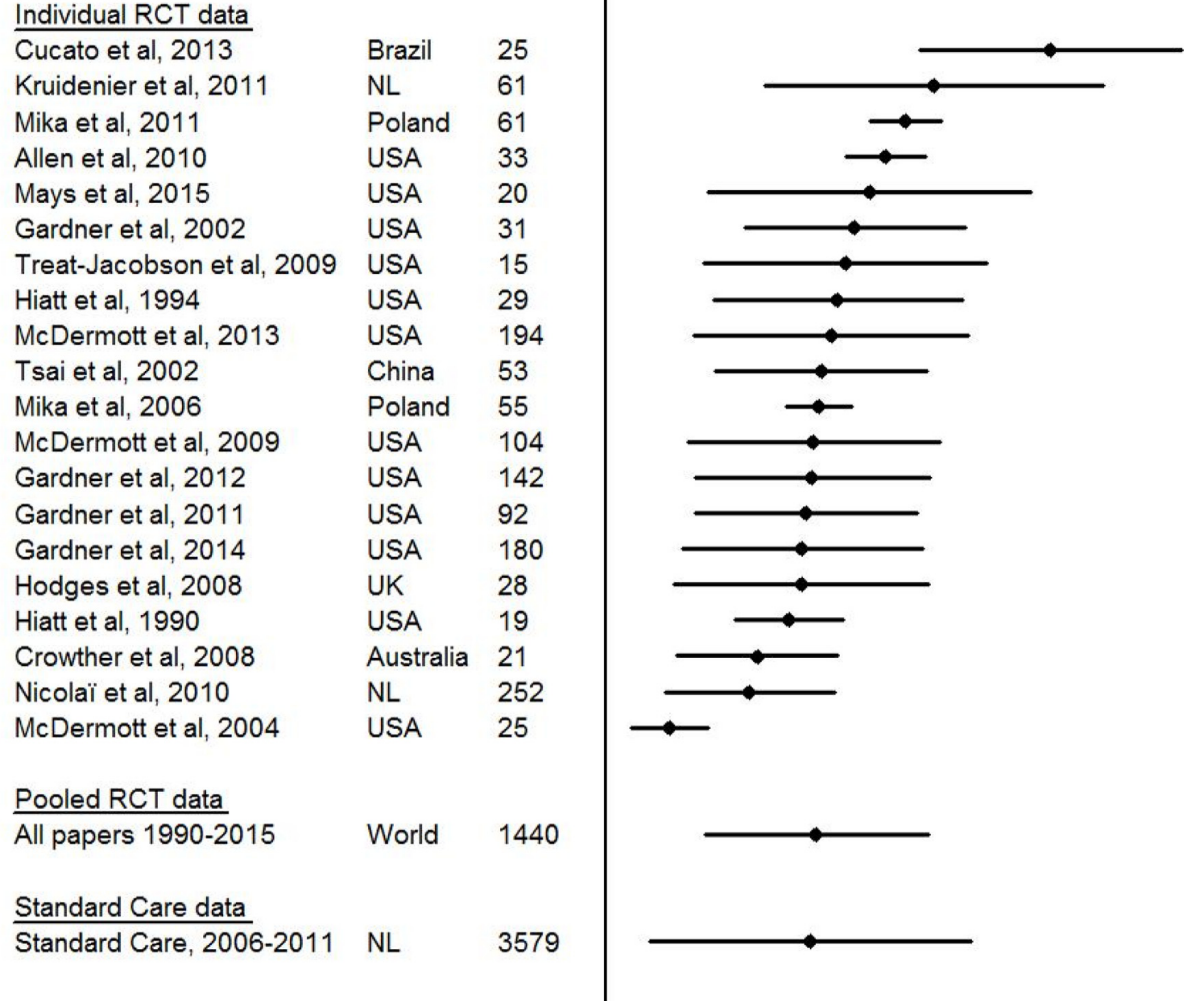
Forest plot illustrating the mean (SD) baseline maximum walking distance of patients included in the 20 RCTs and patients treated in standard supervised exercise therapy care.

Mean baseline maximum walking distance was 382 m (range 116 to 805 m) in the 20 RCTs. No difference in baseline maximum walking distance between RCTs with a three months and/or six- months follow up period was found (375m, SD: 218m versus 370m, SD: 216m). In the standard supervised exercise therapy care population mean baseline maximum walking distance was 10 m less; this difference was not clinically relevant (3.14%) nor statistically significant (*p* = 0.232). Thirteen of the 20 RCTs reported higher mean maximum walking distance at baseline than in standard supervised exercise therapy care (Fig 2).

### Comparison of treatment response

Maximum walking distance after three months was studied in 65% and after six months in 35% of all RCTs. After three months maximum walking distance in RCTs was on average 605 m (range: 178 to 1100 m) and after six months 641 m (range: 610 to 956 m). After three and six months, improvement in maximum walking distance was greater in patients treated with supervised exercise therapy in standard physiotherapy care compared to patients included in RCTs (58 m more improvement after 3 months and 159 m more improvement after 6 months). After three and six months of supervised exercise therapy, the difference in improvement in maximum walking distance between patients included in RCT and patients from standard care was statistically significant (both *p* < 0.01). A clinically relevant difference (≥15%) between patients included in RCTs and patients treated in standard care was only found after six months of supervised exercise therapy (maximum walking distance improvement in patients treated in standard care was 24.80% higher). After three months of supervised exercise therapy the improvement in maximum walking distance was 14% higher in patients treated in standard physiotherapy care compared to patients included in RCTs and thus borderline clinically relevant (Table 2).

### Sensitivity analysis

In some RCTs researchers used different treadmill protocols to assess maximum walking distance [44, 45] we found, however, that this did not result in differences in maximum walking distance assessed at baseline.

## Discussion

All RCTs included in the present study excluded moderate to large segments of the population of patients with intermittent claudication, and the exclusion criteria applied were justified in only a third of the trials. It seems that most of the exclusion criteria are also implicitly used in standard physiotherapy care, because baseline characteristics of patients included in RCTs and patients treated in standard physiotherapy care were comparable. However, a statistically significant and (borderline) clinically relevant difference in the improvement in maximum walking distance after three and six months was found. The difference between trial treatment response and standard physiotherapy care treatment response suggests an underestimation of the benefits of supervised exercise therapy for the standard physiotherapy care setting.

The strengths of our study include the rigorous literature search, the data extraction check and the availability of a large real-life standard care supervised exercise therapy data registry. There are also some limitations. First, we did not have data on the exclusion criteria used in standard physiotherapy care, making a head-on comparison impossible. Second, due to a lack of disease-specific reporting of comorbidity in the standard care data, we could not compare patients included in trials exactly with patients treated in standard supervised exercise therapy care with regard to disease-specific comorbidity. Third, no information on possible supervised exercise therapy adaptations applied in standard physiotherapy care was available, nor did any of the RCTs provide treatment fidelity estimates. Finally, we had no control over how well standard supervised exercise therapy care data was recorded. Nevertheless, data on 3,531 patients was collected by 197 different physiotherapists and therefore we are inclined to accept that quality differences will balance out due to the large number of physiotherapists registering the data.

Our inventory of eligibility criteria and justifications for eligibility criteria used in RCTs investigating the effect of supervised exercise therapy in patients with intermittent claudication showed that all trials excluded large segments of the population of patients with intermittent claudication, yet this did not result in major differences at baseline. Patients were excluded due to comorbidity in all 20 RCTs. Cardiovascular comorbidity was the reason for exclusion in 85% of the RCTs and only one article justified this exclusion criteria, stating that cardiac symptoms might limit walking ability of patients with intermittent claudication to a greater extent than peripheral arterial disease [33]. Coronary artery disease was shown to be present in 46% to 58% of all patients with intermittent claudication [46, 47], which implies that the exclusion of patients with this comorbidity leaves half of patients with intermittent claudication with uncertainty and potential unintended harm from generalizing trial results. Due to the fact that RCTs have limited financial resources, follow a strict intervention protocol and cardiac exercise testing would be recommended before the start of supervised exercise therapy, [11] the exclusion of patients with cardiovascular comorbidity seems understandable; however, this has the consequence of limiting the generalizability of the RCTs results. Patients who received invasive treatment in the preceding six months or were scheduled for an invasive intervention were also often excluded. McDermott et al. [33] excluded this patient group because lower extremity revascularization may alter walking ability. This justification is in line with previous research that has shown that invasive treatment is effective in increasing maximum walking distance at a follow-up to 12 months [48, 49]. Medication use (unstable and in general) as well as impairments in walking capacity were also reported as exclusion criteria in the majority of included RCTs, again with limited justification. A rationale for the eligibility criteria used is hardly ever provided, although international ethical guidelines for research involving human subjects [50, 51] and the CONSORT and the SPIRIT statement require justification of exclusion of study populations [52, 53]. Gardner et al. [26] did not explicitly report rationales for exclusion, but provided references [42, 43] as support for the exclusion criteria applied. However, these references are complex documents that keep the reader guessing about the exact reasons why patients have been deliberately excluded. The justification for exclusion criteria applied was made transparent on a more general level; hence explicit reporting is necessary to study concerns of unjustifiable exclusion of the study population. None of the 20 RCTs reported how many patients were excluded per eligibility criterion. Regardless, we also found that the majority of baseline characteristics of patients with intermittent claudication included in RCTs and patients with intermittent claudication treated in standard care were comparable (age, BMI, smoking behavior, comorbidity and maximum walking distance). We did observe a higher number of male patients in the RCTs, but we are unable to formulate a logical rationale for this difference. Our a-priori hypothesis that baseline maximum walking distance would be lower in standard supervised exercise therapy practice was thus rejected. This leads us to assume that patients with certain types of comorbidity are also not part of standard practice. There might be multiple underlying reasons. It could be that the impact of comorbidity on walking ability, exercise capacity, its severity or in general the presence of comorbidity discourages patients from seeking physiotherapy treatment. Previous research showed that comorbidity may require adaptations in physiotherapy as comorbidity negatively affects treatment outcomes of the index disease or as treatment for one disease may negatively interact with the treatment or natural course of the comorbidity [54]. It might also be that physiotherapists recognize additional risks of exercise training that emerge due to comorbidity and therefore advise against supervised exercise therapy [11]. Whatever the reason, we conclude that the degree of selection bias in RCTs on supervised exercise therapy in people with intermittent claudication is minimal and would therefore likely reflect the general population of individuals with intermittent claudication.

Improvement in maximum walking distance after three and six months of supervised exercise therapy was significantly greater–both statistically as well as clinically–for patients treated in standard physiotherapy care compared to patients treated in RCTs, refuting our a-priori hypothesis. Considering that baseline maximum walking distance values were comparable, this difference might be attributed to differences in the utilized supervised exercise therapy treatment protocols. In general, patients in the RCTs and in standard care received supervised exercise therapy on a treadmill, three times a week at a walking speed of 3.2 km/h, except for the trial of McDermott et al. [35] where patients walked around an indoor track. Despite the fact that exercise duration per treatment session was on average 12 minutes longer in RCTs than in standard care, no striking differences between the description of supervised exercise therapy as applied in the RCTs and in standard care were found that explain this substantial disparity in treatment response; both largely follow the current recommendations for optimal supervised exercise therapy [55–57]. In our opinion, there are two possible explanations for the differences in treatment response: the therapeutic quality and fidelity of the provided interventions and differences in utilized methods. First, we expect that the therapeutic quality of the supervised exercise therapy interventions in standard practice is greater in comparison to supervised exercise therapy in RCTs. After all, specialized and well-trained therapists have considerable experience providing supervised exercise therapy to people with intermittent claudication, likely resulting in more personalized therapeutic interventions, as compared to the often rigid and highly standardized interventions provided in RCTs [58]. Following a highly standardized supervised exercise therapy protocol might cause the ‘better patients’ to be insufficiently challenged, while in standard care supervised exercise therapy treatment protocols might have been tailored to the individual patient circumstances in order to gain the maximum improvement in maximum walking distance. A fully detailed description of the intervention applied in RCTs is recommended by the Consolidated Standards of Reporting Trials (CONSORT) [51]. All RCTs included in the present study present a moderate level of detail about supervised exercise therapy. Although, it might not be possible to present a great level of detail in scientific articles, we support the suggestion by Schulz et al. [59] to post links to websides that then provide a detailed description of the intervention applied in the RCT.

Moreover, trial participants may have been limited in their choice of possible co-interventions; after all, co-intervention is typically seen as a potential source of bias in RCTs. In contrast, patients with intermittent claudication who are referred to standard physiotherapy care for supervised exercise therapy will likely receive additional therapeutic recommendations, home-exercise programs and perhaps even treatment, which in turn could lead to a greater improvement in maximum walking distance over time.

Moreover, it is important to highlight that data from standard physiotherapy care was collected between 2006 and 2011 and SET was back then in its infancy [60, 61]. Based on the positive effect of SET, in 2011 a nationwide community based SET network (ClaudicatioNet) was initiated and launched in the Netherlands. The forefront of ClaudicatioNet is formed by specialised physical therapists providing high quality SET, stimulating lifestyle changes and medication adherence [62, 63]. Due to the progress in quality of SET care we speculate that repeating the present study with more recent standard care data might result in even a larger discrepancy between SET effectiveness presented in RCTs and SET applied in standard care.

Our assessment of the methodological quality of RCTs included showed that in all RCTs bias occurred because of lack of blinding and intention-to-treat analysis. Blinding is a critical methodological part of RCTs. However, the results of our quality assessment indicated that 100% of the included RCTs did not blind subjects and therapists and that the assessor was blinded in only a quarter of the studies. A number of studies previously reported a significant difference in the size of the estimated treatment effect between trials that reported blinding compared to those that did not (p = 0.01), with an overall odds ratio 17% larger in studies that did not report ‘double-blinding’ [64–66]. Because none of the 20 included RCTs reported ‘double-blinding’, their estimated treatment effect represents an underestimation (-17%) of treatment effectiveness in ideal methodological circumstances. This means that our RCT data is more comparable to the standard care data (no blinding), and that even despite the fact that the estimate of treatment effectiveness in RCTs is underestimated by 17% a statistical and (borderline) clinically relevant difference in treatment effectiveness has been detected after three and six months. Moreover, blinding of the outcome assessor is crucial to ensure unbiased ascertainment of treatment effectiveness [67]. However, the lack of blinding techniques that has been detected in the majority of RCTs included in the present study also enhances the comparability with our standard care data.

Less than half of all included RCTs performed an intention-to-treat analysis (ITT), which has been proven to be the least biased technique to estimate intervention effects in RCTs [68]. This is because an ITT tests the hypothesis of a null effect of treatment in a statistically valid way, and if participants do not fully adhere to their assigned treatment an ITT underestimates the treatment effect [69]. To maintain the benefits of randomization, ITT requires the comparison to be based upon the treatment and control groups participants were randomly assigned to. In contrast, per-protocol analysis describes the outcomes of the participants who adhered to the research protocol, which can be useful to estimate the intervention’s efficacy for those who actually received it. Nevertheless, this estimate is likely to be flawed (overestimated) due to nonadherence of participants [68]. Likewise, this also means that the treatment effect might be overestimated in 11 RCTs.

Methodological differences between the RCT study design and our analysis of data from standard care physiotherapy must also be discussed. Overall, the main difference between our comparison of RCTs and observational standard care data is the fact that in the RCTs, supervised exercise therapy is randomly allocated to the patients, whereas in standard care patients are naturally exposed to supervised exercise therapy. In the latter, voluntarily going to the physiotherapist for supervised exercise therapy might be a characteristic of some patients and these patients might be different from those who do not voluntarily seek help by their physiotherapist for the treatment of intermittent claudication. It might be that patients with intermittent claudication who chose physiotherapy are healthier than patients with intermittent claudication that chose not to go to the physiotherapist. It might also be the case that the patients who receive supervised exercise therapy in the context of standard care have been chosen by someone else, such as the vascular surgeon who referred them to the physiotherapist for supervised exercise therapy. This possibly existing imbalance, which could also be called selection bias or referral bias, might have led to an overestimation of maximum walking distance improvement after supervised exercise therapy [70]. Furthermore, treatment compliance may not be equivalent between the RCT and the standard care setting. The correctness of data entry in the EMR might differ from the correctness of data entry from the RCT, because standard care data is recorded within the workflow of daily practice and RCTs are often based on double-data entry and data checks. Although there are of course discrepancies between both settings that are the result of biological and methodological differences, a systematic review published in the New England Journal of Medicine concluded that observational studies did not systematically overestimate the magnitude outcomes as compared with results of RCTs [70].

## Conclusion

All RCTs included in the present study excluded moderate- to large segments of the population of patients with IC and exclusion criteria applied were justified in only one-third of the trials. Most of the exclusion criteria are also implicitly used in standard physiotherapy care and because of this baseline characteristics of patients included in RCTs and patients treated in standard physiotherapy care were comparable. However, a statistically significant and (borderline) clinically relevant difference in the improvement in MWD after three and six months was found. The difference between trial treatment response and standard physiotherapy care treatment response suggests an underestimation of the benefits of SET for the standard physiotherapy care setting. Moreover, the results of the present study might stimulate researchers and health policy advisors to reconsider which study design serves best to communicate the effectiveness of an intervention to stakeholders.

## Supporting Information

S1 PRISMA Checklist PRISMA Checklist. (DOC)

S1 Table Search string as utilized in Pubmed. (DOCX)

S2 Table Categories of comorbidity (cohort data). (DOCX)

S3 Table Methodological Quality of RCTs. (DOCX)

S4 Table Comparison of comorbidity. (DOCX)

S5 Table Justification of exclusion criteria. (DOCX)

S6 Table Treadmill testing protocols. (DOCX)
