# Supplementary material for: How Well Do Randomized Controlled Trials Reflect Standard Care: A Comparison between Scientific Research Data and Standard Care Data in Patients with Intermittent Claudication undergoing Supervised Exercise Therapy
Source: PLoS One. 2016 Jun 23;11(6):e0157921. doi: 10.1371/journal.pone.0157921 (PMC4919097; doi:10.1371/journal.pone.0157921)
Supplement: S1 Table — (DOCX) [file pone.0157921.s002.docx]

**S1 Table.**

**Search string as utilized in Pubmed.**

#1 Peripheral Arterial Disease [MeSH Terms] OR Arterial Occlusive Diseases [MeSH Terms] OR Intermittent Claudication [MeSH Terms]

#2 Peripheral Artery Disease [Text Word] OR Peripheral Artery Diseases [Text Word] OR Peripheral Arterial Disease [Text Word] OR Peripheral Arterial Diseases [Text Word] OR Arterial Occlusive Disease [Text Word] OR Artery Occlusive Disease [Text Word] OR Arterial Obstructive Disease [Text Word] OR Artery Obstructive Disease [Text Word] OR Intermittent Claudication [Text Word]

#3 Exercise [MeSH Terms] OR Exercise Therapy [MeSH Terms] OR Physical Therapy Modalities [MeSH Terms]

#4 exercise [Text Word] OR walk* [Text Word] OR treadmill [Text Word] OR physical training [Text Word]

#5 Search #1 OR #2

#6 Search #3 OR #4

#7 Search #5 AND #6
