# Supplementary material for: How Well Do Randomized Controlled Trials Reflect Standard Care: A Comparison between Scientific Research Data and Standard Care Data in Patients with Intermittent Claudication undergoing Supervised Exercise Therapy
Source: PLoS One. 2016 Jun 23;11(6):e0157921. doi: 10.1371/journal.pone.0157921 (PMC4919097; doi:10.1371/journal.pone.0157921)
Supplement: S2 Table — (DOCX) [file pone.0157921.s003.docx]

**S2 Table.**

**Categories of comorbidity (cohort data)**

**Cardiac comorbidity** included the presence of:

Hypertension, abnormal electrocardiography, abnormal sinus rhythm, abnormal functional capacity, angina pectoris, heart failure, myocardial infarction, coronary arterial bypass graft, percutaneous transluminal coronary angioplasty, rhythm disorders, cardiac decompensation, heart valve suffering, PTA, recanalization, bypass operation, endarterectomy, hypertension, arrhythmia, cardiac decompensation, heart valve suffering and other cardiovascular pathologies.

**Pulmonary comorbidity** was defined as having:

Asthma, interstitial lung disease, diffuse interstitial lung disease / sarcoidosis, emphysema, cystic fibrosis, sleep apnea, hyperplasia / malignancy, chronic obstructive pulmonary disease and other pulmonic disorders.

**Internal comorbidity** involved:

Hypercholesterolemia, hyperlipidemia, elevated homocysteine ​​levels, abnormal lipid spectrum, renal impairment, obesity, hyperplasia / malignancy, immunity disorder, diabetes mellitus and other internal pathologies.

**Orthopedic comorbidity** contained of:

Arthritis, rheumatoid arthritis, polyarthritis, osteoporosis, joint prosthesis, arthrogenic impairment, congenital dysplasia, congenital scoliosis, M. Scheuerman, M. Bechterew, fractures, hyperplasia / malignancy, Reiter's syndrome, Systemic Lupus Erythematous (SLE), psoriasis, polymyositis and other orthopedic disorders were described to have.

**Neurologic comorbidity** was present in patients with:

Cerebrovascular accident / a transient ischemic attack / central paresis, peripheral nerve disease, cerebellar disorders / encephalopathy, multiple sclerosis / amyotrophic lateral sclerosis / spinal cachexia, Parkinson / Extrapyramidal disorders, hernia nuclei pulposi with motor loss, radiculopathy / radicular syndrome, Paraplegics (traumatic / partial), neurotrauma and hyperplasia / malignancy.
