# Supplementary material for: How Well Do Randomized Controlled Trials Reflect Standard Care: A Comparison between Scientific Research Data and Standard Care Data in Patients with Intermittent Claudication undergoing Supervised Exercise Therapy
Source: PLoS One. 2016 Jun 23;11(6):e0157921. doi: 10.1371/journal.pone.0157921 (PMC4919097; doi:10.1371/journal.pone.0157921)
Supplement: S3 Table — (DOCX) [file pone.0157921.s004.docx]

**S3 Table.**

**Methodological Quality of RCTs.**

|  | Eligibility criteria | Random allocation | Concealed allocation | Baseline comparability | Blind subjects | Blind therapists | Blind assessors | Adequate follow-up | Intention-to treat analysis | Between-group comparisons | Point estimates and variability | Total* |
| --- | --- | --- | --- | --- | --- | --- | --- | --- | --- | --- | --- | --- |
| Allen et al, 2010 | Yes | Yes | No | Yes | No | No | No | No | No | Yes | Yes | 4/10 |
| Crowther et al, 2008 | Yes | Yes | No | Yes | No | No | No | Yes | No | Yes | Yes | 5/10 |
| Cucato et al, 2013 | Yes | Yes | No | Yes | No | No | No | Yes | No | Yes | Yes | 5/10 |
| Gardner et al  2002 | Yes | Yes | No | Yes | No | No | No | No | Yes | Yes | Yes | 5/10 |
| Gardner et al, 2011 | Yes | Yes | Yes | Yes | No | No | No | No | No | Yes | Yes | 5/10 |
| Gardner et al, 2012 | Yes | Yes | No | Yes | No | No | No | No | Yes | Yes | Yes | 5/10 |
| Gardner et al, 2014 | Yes | Yes | No | Yes | No | No | No | Yes | Yes | Yes | Yes | 6/10 |
| Hiatt et al, 1990 | No | Yes | No | Yes | No | No | No | No | No | Yes | Yes | 4/10 |
| Hiatt et al, 1994 | No | Yes | No | Yes | No | No | No | Yes | No | Yes | Yes | 5/10 |
| Hodges et al, 2008 | Yes | Yes | No | No | No | No | No | No | No | Yes | Yes | 3/10 |
| Kruidenier et al, 2011 | Yes | Yes | Yes | Yes | No | No | No | Yes | Yes | Yes | Yes | 7/10 |
| Mays et al, 2015 | Yes | Yes | Yes | Yes | No | No | No | Yes | Yes | Yes | Yes | 7/10 |
| McDermott et al, 2004 | Yes | Yes | No | Yes | No | No | No | No | Yes | Yes | No | 4/10 |
| McDermott et al, 2009 | Yes | Yes | No | Yes | No | No | Yes | Yes | Yes | Yes | Yes | 7/10 |
| McDermott et al, 2013 | Yes | Yes | No | Yes | No | No | Yes | Yes | Yes | Yes | Yes | 7/10 |
| Mika et al, 2006 | Yes | Yes | No | Yes | No | No | Yes | Yes | No | Yes | Yes | 6/10 |
| Mika et al, 2011 | Yes | Yes | No | Yes | No | No | Yes | Yes | No | No | Yes | 5/10 |
| Nicolaï et al, 2010 | Yes | Yes | Yes | Yes | No | No | Yes | No | No | Yes | Yes | 6/10 |
| Treat-Jacobson et al, 2009 | Yes | Yes | No | Yes | No | No | No | Yes | No | Yes | Yes | 5/10 |
| Tsai et al, 2002 | Yes | Yes | No | Yes | No | No | No | No | Yes | Yes | Yes | 5/10 |

**Table 1.** Methodogical quality of all randomized controlled trials included.
