# Supplementary material for: How Well Do Randomized Controlled Trials Reflect Standard Care: A Comparison between Scientific Research Data and Standard Care Data in Patients with Intermittent Claudication undergoing Supervised Exercise Therapy
Source: PLoS One. 2016 Jun 23;11(6):e0157921. doi: 10.1371/journal.pone.0157921 (PMC4919097; doi:10.1371/journal.pone.0157921)
Supplement: S4 Table — (DOCX) [file pone.0157921.s005.docx]

**S4 Table.**

**Comparison of comorbidity.**

**Table 1.** Number of patients with a disease-specific comorbidity reported in RCTs and approximate estimate (worst and best case scenario) of the percentage patients included in RCTs with a comorbidity per category compared to the percentage patients with a comorbidity per category from standard physiotherapy care.

|  |  | Allen 2010 | Crowther 2008 | Cucato 2013 | Gardner 2011 | Gardner 2012 | Hiatt 1994 | Kruidenier 2011 | McDermott 2004 | McDermott 2009 | McDermott 2013 | Nicolai 2010 | Treat-Jacobson 2009 | Total RCTs | Standard care^a^ |
| --- | --- | --- | --- | --- | --- | --- | --- | --- | --- | --- | --- | --- | --- | --- | --- |
|  | Total N | N=76 | N=21 | N=25 | N=80 | N=142 | N=29 | N=70 | N=32 | N=103 | N=194 | N=304 | N=25 | N=1,101 | N=3,541 |
|  | N CAD | 14 |  |  |  |  | 9 |  | 5 |  | 30 |  |  |  | 1983/3541 (= 56%) |
|  | N CHF | 5 |  |  |  |  |  | 20 |  |  | 22 | 76 | 14 |  |  |
| Cardiovascular | N High BP |  | 7 | 21 | 70 | 91 | 14 | 49 |  |  |  |  | 16 |  |  |
|  | N IHD |  | 5 |  |  |  |  |  | 5 |  |  |  |  |  |  |
|  | Worst case | 19 | 12 | 21 | 70 | 91 | 23 | 69 | 10 |  | 52 | 76 | 30 | 473/998 (= 47%) |  |
|  | Best case | 14 | 7 | 21 | 70 | 91 | 14 | 49 | 5 |  | 30 | 76 | 16 | 393/998 (= 39%) |  |
|  | | | | | | | | | | | | | | | |
| Pulmonary | N COPD |  |  |  |  |  | 4 | 8 | 2 |  | 26 | 63 |  |  | 637/3541 (=18%) |
|  | Worst and best case |  |  |  |  |  | 4 | 8 | 2 |  | 26 | 63 |  | 103/629 (=16%) |  |
|  | | | | | | | | | | | | | | | |
|  | N DM |  | 4 | 5 | 34 | 35 |  | 14 | 12 | 44 | 64 | 69 | 7 |  | 1912/3541 (=54%) |
|  | N Dyslip. |  |  | 23 | 71 | 83 | 7 |  |  |  |  |  | 19 |  |  |
|  | N MS |  |  |  | 62 | 78 |  |  |  |  |  |  |  |  |  |
|  | N Chol↑. |  |  |  |  |  |  | 61 |  |  |  |  |  |  |  |
| Internal | N Cancer |  |  |  |  |  |  |  |  |  | 31 |  |  |  |  |
|  | Worst case |  | 4 | 28 | 167 | 196 | 7 | 75 | 12 | 44 | 95 | 69 | 26 | 723/1025 (=71%) |  |
|  | Best case |  | 4 | 23 | 71 | 83 | 7 | 61 | 12 | 44 | 64 | 69 | 19 | 457/1025 (=45%) |  |
|  | | | | | | | | | | | | | | | |
|  | N OA |  | 3 |  |  |  |  | 12 | 8 |  | 44 | 47 |  |  | 850/3541 (=24%) |
|  | N Spinal S |  |  |  |  |  |  |  |  |  | 20 |  |  |  |  |
| Orthopedic | Worst case |  | 3 |  |  |  |  | 12 | 8 |  | 64 | 47 |  | 134/621 (=22%) |  |
|  | Best case |  | 3 |  |  |  |  | 12 | 8 |  | 44 | 47 |  | 114/621 (=18%) |  |
|  | | | | | | | | | | | | | | | |
| Neurologic | N CVA | 8 |  |  |  |  | 6 | 6 | 3 |  | 24 | 38 |  |  | 850/3541 (=24%) |
|  | Worst and best case | 8 |  |  |  |  | 6 | 6 | 3 |  | 24 | 38 |  | 85/705 (=12%) |  |
| ^a^Detailed information about which diseases belong to each category of comorbidity in standard care can be found in Appendix 1. Abbreviations: CAD: coronary artery disease; CHF: congestive heart failure; High BP: high blood pressure (Hypertension); IHD: Ischemic heart disease; COPD: chronic obstructive pulmonary disease; DM: diabetes mellitys; Dyslip: Dyslipidemia; MS: metabolic syndrome; Chol.↑: hypercholesterolemia; OA: osteoarthritis; Spinal S: Spinal stenosis; CVA: cerebrovascular disease | | | | | | | | | | | | | | | |
