# Supplementary material for: How Well Do Randomized Controlled Trials Reflect Standard Care: A Comparison between Scientific Research Data and Standard Care Data in Patients with Intermittent Claudication undergoing Supervised Exercise Therapy
Source: PLoS One. 2016 Jun 23;11(6):e0157921. doi: 10.1371/journal.pone.0157921 (PMC4919097; doi:10.1371/journal.pone.0157921)
Supplement: S5 Table — (DOCX) [file pone.0157921.s006.docx]

**S5 Table.**

**Justification of exclusion criteria.**

**Gardner et al. [26]:**

Guidelines of the American college of Sports Medicine [[42](#_ENREF_42)] and a study conducted by Regensteiner et al. [[43](#_ENREF_43)] were mentioned as justification.

**Hiatt et al. [29]:**

Patients with diabetes mellitus were excluded because (1) the response to a conditioning program may be affected by glycemic control and (2) sever and distal distribution of arterial occlusive disease is often present in diabetic patients. As drugs may affect exercise training, patients using β-adrenergic-blocking drugs or pentoxifylline were excluded.

**Hiatt et al. [30]:**

Patients with diabetes mellitus were excluded because glycemic control may affect their response to exercise.

**McDermott et al. [33]:**

Men and women with symptoms of IC were excluded because the aim of this study was to study the feasibility of SET for symptom-free PAD patients. The following participants were considered to not likely complete a 12-week SET program three times per week at the medical center and were therefore excluded: (1) individuals with above-knee and below- knee amputation; (2) individuals with a wheelchair; (3) individuals that are unable to walk without a cane or walker; (4) individuals unable to come to the medical center three times per week and (5) individuals who received cancer treatment during the preceding 6 months. As lower extremity revascularization may alter walking ability individuals scheduled for it were excluded. Individuals with angina pectoris of condition that exceeds class 2 New York Heart Association heart failure were excluded because this cardiac symptoms might limit walking ability to a greater degree than PAD. Individuals with chronic obstructive pulmonary disease (COPD) were included to enhance the generalizability of the results. For the same reason no restrictions on blood pressure or diabetes control were listed.

**Mika et al. [36]:**

Because there might be an effect of drugs on exercise training, patients using β-adrenergic-blocking drugs, pentoxifylline or other hemorheologically active drugs were excluded.

**Mika et al. [37]:**

Menopausal women and women using estrogen were excluded because both might influence high-density lipoprotein cholesterol (HDL-C) concentration

None of the other RCTs did provide justification for eligibility criteria applied.
