# Supplementary material for: How Well Do Randomized Controlled Trials Reflect Standard Care: A Comparison between Scientific Research Data and Standard Care Data in Patients with Intermittent Claudication undergoing Supervised Exercise Therapy
Source: PLoS One. 2016 Jun 23;11(6):e0157921. doi: 10.1371/journal.pone.0157921 (PMC4919097; doi:10.1371/journal.pone.0157921)
Supplement: S6 Table — (DOCX) [file pone.0157921.s007.docx]

**S6 Table.**

**Treadmill testing protocols.**

**Gardner et al. [44]:**

Subjects walk on the treadmill at an initial workload of 3.2 km/h. Subjects start with 0% incline for the first 2 minutes, which is then increased by 2% every 2 minutes without change in speed.

**Hiatt et al. [45]:**

Subjects walk on the treadmill at an initial workload of 2 mph (3.2km/h), 0% grade for 3 minutes. Subsequent stages increased 3.5% in grade every 3 minutes without change in speed.

**Treat- Jacobson [39]:**

Graded cardiopulmonary treadmill test (GXT): the GXT protocol consisted of walking on the treadmill at a speed of 2 mph (3.2km/h) at 0% grade. The treadmill grade was increased 3.5% every 3 minutes until a 10.5% grade was obtained, at which the speed was increased by 0.5 mph (0.8km/h) every 3 minutes, while maintaining the grade at 10.5%.
